# Nurse led versus lay educators support for those with asthma in primary care: a costing study

**DOI:** 10.1186/1471-2466-12-52

**Published:** 2012-09-08

**Authors:** Nicola J Roberts, Kathleen A Boyd, Andrew H Briggs, Ann L Caress, Martyn R Partridge

**Affiliations:** 1 Institute for Applied Health Research/School of Health and Life Sciences Glasgow Caledonian University, Glasgow, UK; 2 Health Economic and Health Technology Assessment, Institute of Health & Wellbeing University of Glasgow, Glasgow, UK; 3 Nursing, Midwifery and Social Work, University of Manchester, Manchester, UK; 4 NHLI Division at Charing Cross Hospital, Imperial College, London, UK

## Abstract

**Background:**

Regular review and support for asthma self-management is promoted in guidelines. A randomised controlled trial suggested that unscheduled health care usage was similar when patients were offered self management support by a lay-trainer or practice nurses.

**Methods:**

Following the RCT, a costing study was undertaken using the trial data to account for the cost of delivery of the service under both strategies and the resulting impact on unscheduled healthcare (measure of effectiveness) in this trial.

**Results:**

One year data (n = 418) showed that 29% (61/205) of the nurse group required unscheduled healthcare (177 events) compared with 30.5% (65/213) for lay-trainers (178 events).

The training costs for the lay-trainers were greater than nurses (£36 versus £18 respectively per patient, p<0.001), however, the consultation cost for lay-trainers were lower than nurses (£6 per patient versus £24, p<0.001). If the cost of unscheduled healthcare are accounted for then the costs of nurses is £161, and £135 for lay-trainers (mean difference £25, [95% CI = −£97, £149, p = 0.681]). The total costs (delivery and unscheduled healthcare) were £202 per patient for nurses versus £178 for lay-trainers, (mean difference £24, [95%CI = −£100, £147, p = 0.707]).

**Conclusions:**

There were no significant differences in the cost of training and healthcare delivery between nurse and lay trainers, and no significant difference in the cost of unscheduled health care use.

## Background

Worldwide, 300 million people have asthma [1] and within the UK there are approximately 5.2 million people with the condition. National [2] and international guidelines [3] document the importance of self management education which allow patients to manage their own asthma and educate patients to recognise when their symptoms are worsening as well as how and when to get emergency care. There have been many studies looking at the effectiveness of self-management education, support and regular review including a Cochrane review [4]. Several studies have shown that an important component of the education is the receipt by patients of a written personalised asthma action plan [5].

In the UK asthma is predominately managed in primary care where it is usually provided by practice based nurses than by GPs. Written personalised asthma action plans have been recommended in National guidelines for 20 years [6,7] but studies have shown that between 3-20% of patients have such personalised asthma action plans. There are many reasons why nurses and other health professionals fail to implement guideline recommendations [8], and this has been well documented in the literature [9-13]. These include lack of self-motivation and experience of self management [8,11-13], lack of appropriate materials [14] and unclear roles relating to self management [10,12].

If implementation of guideline recommendations remains sub-optimal, alternatives to health professional delivery of patient review and self management support needs to be investigated, such alternatives include consultation aids or tools such as educational leaflets, videos [15-19] or computer software [20]. Alternatively lay people can be trained to deliver self management advice freeing nurses and other health professionals’ time to perform other clinical duties [21-23]. It is important that any changes to the delivery of care are clinically as effective as the traditional method of care, (in this case by nurses). It is also important to consider potential cost implications.

The Expert Patient Programme [24] has shown that lay people, with good training and support, can make excellent facilitators. In a study by Partridge et al. [22] lay educators were either individuals with asthma or those who had a family member with asthma. The study showed that lay trainers were as effective as nurses in reviewing patients and offering self management support but within the trial timeline the costs were not assessed. The study was a randomised equivalence trial designed to test the hypothesis that well trained lay people could offer review and self-management support to adults with asthma with results which would be equivalent to nurse led education. The implicit assumption was that lay educators would be cheaper than nurses and could be used potentially to free up nurse time. This costing study sets out to calculate the within trial costs and present the unscheduled healthcare usage outcomes and associated costs to determine whether the use of lay educators is a financially viable alternative to usual care (delivery by primary care based practice nurses).

## Methods

### Trial procedures

The trial was undertaken in 2004/5 with the fully informed consent of all participants. A detailed description of the trial procedures are published elsewhere [22]. A randomised equivalence trial was undertaken to assess whether well trained lay people could offer review and self-management support to adults with asthma with results which would be equivalent to nurse led care. Patients were recruited at two locations: West London and North West England. In total 567 patients were randomised to either a lay educator or a practice based nurse within each participating general practice. Eligible patients were adults aged 18 or over with clinician diagnosed asthma with persistent disease requiring regular preventative therapy. Participants also had evidence of unscheduled health care usage or increased medication for the treatment of an exacerbation in the 12 months prior to recruitment. The intervention was a disease specific asthma self management programme delivered by the practice nurse or lay educator. A total of 19 lay educators were recruited, who had personal or family experience of asthma. 15 of these were trained however only 8 actively participated in the trial. Lay educators were involved in the trial on a part-time basis and were not contractually employed. Both the lay educators and the practice nurses received specialised training prior to the trial. The lay educators received a 2 day Education for Health training course (http://www.educationforhealth.org/) with follow-up distance learning and three 1 day training sessions and on the job mentoring. The 46 practice nurses undertook a 1 day NRTC update programme and received specific training on writing action plans.

The protocol intervention for both the lay educators and nurses offered patients two face-to-face consultations plus follow up support. The first consultation involved a semi structured review of their condition including individualised advice regarding self management summarised in a written personalised asthma action plan. This was followed by a second reinforcing session of up to 30 minutes three weeks after the first appointment. Follow up telephone consultations were carried out by either the practice nurse or lay educator depending on the allocated intervention arm and were undertaken every three months for one year. Topics addressed during consultations were recommended in national and international guidelines and included aetiology of asthma and long term nature of the disease, asthma medications and uses, triggers and allergen avoidance and recognition of worsening symptoms and actions to take. The primary outcome for this study was unscheduled usage of health services (hospital admission, emergency department attendance, unscheduled GP consultation).

Ethical approval for the randomised controlled trial was obtained from the Riverside ethics committee (RREC 3224), and the trial was registered at clinical trials.gov (NCT00129987).

### Cost analysis

#### Cost of delivery and unscheduled healthcare

**Figure 1:**
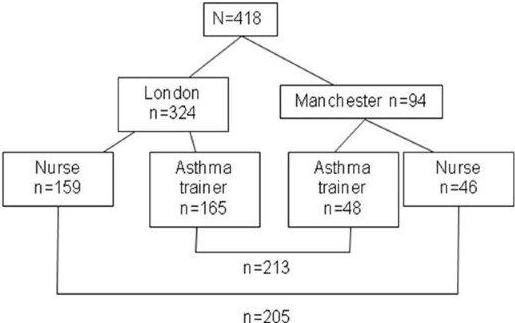
Intention to treat at 1 year – unscheduled use of healthcare n = 418 (Practice nurse arm = 205, lay educators n = 213).

This costing study utilised data from the randomised equivalence trial [23] to compare the training and delivery costs of lay educators versus practice nurses and the effect this has on unscheduled healthcare usage and associated costs for the trial period. The costing evaluation was not carried out alongside the clinical trial but used the trial information retrospectively to undertake a cost analysis, synthesising trial data with that from other sources [25]. Although the trial was carried out at two geographical sites (Figure 1) the baseline analysis was undertaken on the whole dataset and therefore the baseline cost analysis will also be undertaken on the whole dataset. This analysis was undertaken from the NHS perspective and therefore it was not relevant to account for patient costs for travel and time. Resource use and cost information was obtained from the trial data and the Personal Social Services Research Unit (PSSRU) [26]. The outcome data for patient unscheduled health resource use was obtained from the participating general practices.

The costs of the intervention are attributable to two main areas: training, and delivery of self management. The primary outcome (unscheduled healthcare usage) was also incorporated as a cost over a one year duration. All costs are reported in pounds sterling (£) and for price year 2004/2005.

a. Training

Lay educators attended a 2 day residential training course at Education for Health (formerly the National Respiratory Training Council {NRTC}), Warwick followed by a distance learning programme and 3 reinforcing 1 day sessions with an NRTC trainer. Regular monthly mentoring was also incorporated. The cost was invoiced from the NRTC for the whole package of training for each group (London and Manchester) which included venue hire, provision of training materials and training staff. With regards to nurse training, all 46 nurses undertook a 1 day NRTC update programme and received specific training on writing personal asthma action plans and training on the study protocol. Again this was invoiced separately for London and Manchester and included venue, training materials and a trainer. Training costs did not include travel for the lay educators and practice nurses to the training course venues. The costs for training were calculated as a mean cost per randomised patient for each study site and group as shown in Table 1.

**Table 1:** Delivery of training– mean costs per patient

	**Practice nurse (n = 205)**	**Lay educator (n = 213)**
Mean training costs	£18	£36
London	£14	£25
Manchester	£34	£72

b. Delivery

The intervention for both groups consisted of one 45 minute face to face consultation followed by a up to 30 minutes of face to face consultation 3 weeks after the first appointment. The lay educators and nurses carried out the asthma reviews and self management consultations for the first two consultations. Patients then had up to 4 telephone follow-up consultations over the study period from either the practice nurse or lay educator dependant on the intervention arm. The unit cost of practice nurse time for delivering the consultation was calculated using PSSRU reported unit costs [26], and adjusted for higher London costs. This resulted in an hourly unit cost of £28-£32 for practice nurses. The lay educators received an hourly rate of pay, which was £8. The length of the trial consultations were not recorded during the trial, therefore an assumption was made that the consultations lasted as detailed in the protocol for the two face-to-face consultations (1^st^ consultation 45 minutes, 2^nd^ consultation 30 minutes). The cost for the telephone consultations did not include line rental, only the costs per minute for staff to deliver the consultations. The baseline analysis also accounted for the opportunity costs of consultants for those lay trainers who did not claim for their costs. Reimbursement to lay educators for the delivery of consultations was not consistent in the trial as many did not claim for the time they spent carrying out the consultations. The protocol had specified that lay educators would be paid for their time, however, the actual costs incurred were lower than expected due to the lack of claims. The baseline analysis is based on the protocol, and therefore further information was sought from the literature regarding the length of the follow-up telephone consultations so that the cost of lay educators time could be accounted for. Using data from Pinnock et al. [27] an assumption was made for the length of the telephone consultations. An average telephone asthma review is likely to last approximately 11 minutes, including additional time for abortive calls [27], however, this reference provided no indication of whether this time estimate included preparation time. The consultation type and frequency for patients in both arms during the 12 month period were multiplied to calculate the staff costs using the unit cost estimates from published UK sources.

c. Unscheduled healthcare

The primary outcome measure in the trial was need for unscheduled health care over the study period of 12 months. This included primary care consultations with GPs or practice nurses which could include surgery visits, home visits or telephone consultations and also included any out of hours usage. It also included any need for unscheduled secondary care including hospital admissions, attendances at out-patients or accident and emergency departments. The resource use for primary and secondary healthcare usage was identified from patients’ general practice records and Department of Health reference costs were used to obtain costs per patient. The cost of an average hospital admission, a typical GP and standard A&E attendance and a cost of one night in ICU were included. The frequency of each type of unscheduled healthcare was totalled for each patient and multiplied to calculate the cost of unscheduled healthcare using the unit cost estimates. The costs were calculated for each individual patient for each type of healthcare utilisation, and in some cases individual patients had more than one hospital admission or GP attendance.

The costs of this trial were calculated only for the study period (12 months) and therefore no adjustments were required for future discounting. The patient level data from the trial was used to calculate costs.

### Sensitivity analysis-scenario analysis

The baseline analysis was conservative and takes into account the consultations completed by the practice nurses and lay educators which was shown to be overall, less than the 6 consultations per patient (two face-to-face and four telephone consultations) detailed in the protocol. The baseline analysis was undertaken on the combined dataset as a whole, incorporating some assumptions where there was no evidence from the trial, and therefore the analysis is subject to uncertainty [25]. In order to explore this uncertainty and take a closer look at the outcomes, some scenario analyses were undertaken. The impact on the costs of the interventions were explored in four different ‘scenarios’ examining the effect of individual consultations within the intervention, protocol adherence, testing the assumptions made about the length of the telephone consultations and the effect of the two study sites.

#### Scenario 1

The cost of delivering the intervention assuming all possible consultations were delivered to all patients in each arm (i.e. two face-to-face consultations and four telephone consultations for all patients in the trial).

#### Scenario 2

The cost of delivering the intervention assuming all possible consultations were delivered to all patients in each arm (i.e. two face-to-face consultations and four telephone consultations for all patients in the trial) 50% of the cost of consultations for lay educators were included to reflect the impact of the large majority who did not claim back the costs of their time.

#### Scenario 3

The cost of delivering the intervention assuming all possible consultations were delivered to all patients in each arm (i.e. two face-to-face consultations and four telephone consultations). Telephone consultations were costed at 20 minutes to calculate the impact of allowing for preparation time instead of the assumption of 11 minutes.

#### Scenario 4

The cost of delivering the intervention assuming all possible consultations were delivered to all patients in each arm (i.e. two face-to-face consultations and four telephone consultations). Telephone consultations were costed at 20 minutes to allow for preparation time instead of the assumption of 11 minutes. The length of the consultations for the nurses were halved, experienced nurses may have had shorter consultations compared to lay educators. The first consultation was reduced from 45 minutes to 22.5 and the second from 30 minutes to 15 minutes.

### Statistical analyses

Data were analysed using STATA version 10. Mean healthcare costs were calculated and compared using independent t-tests.

## Results

### Cost of intervention

Five hundred and sixty seven patients were randomised to care by a nurse (n = 287) or a lay educator (n = 280). One year data on use of unscheduled healthcare data was available for 418 patients (Practice nurse = 205, Lay educator = 213) as shown in Figure 1. Under an intention to treat approach for those who we had data for at one year, 85/205 (41.5%) in the nurse arm had two face to face consultations (199 face-to-face consultations in total) and 127/213(59.6%) in the lay educator arm (277 face-to-face consultations in total). Patients should have had up to four telephone consultations each but the average per patient was less than one in both nurses and lay trainers. These trial outcomes are presented in Table 2.

**Table 2:** Delivery of the intervention – mean costs per patient

	**Practice nurse (n = 205, London = 159, Manchester = 46)**			**Lay educator (n = 213, London = 165, Manchester = 48)**		
	Cost*	**Quantity delivered**	**Total cost**	**Cost**	**Quantity delivered**	**Total cost (£)**
Face to face 45 mins - London	£24	93	£2232	£6	121	£726
Face to face 45 mins - Manchester	£21	21	£441	£6	29	£174
Face to face 30 mins - London	£16	64	£1024	£4	100	£400
Face to face 30 mins - Manchester	£14	21	£294	£4	27	£108
Telephone consultation - London (11mins)	£6	67	£402	£1	86	£86
Telephone consultation - Manchester (11 mins)	£5	59	£295	£1	98	£98
Total number of consultations		325	£4688		461	£1592

* Consultation costs were taken from PSSRU for practice nurses adjusting for London costs. Lay educator costs were taken from within trial costs.

### Training

The total cost of training the lay educators was £10,368, with a mean cost of £36 per patient. The mean costs per randomised patient were higher at the Manchester site (£72) than the London site (£25), due to lower patient numbers at the Manchester site but similar training costs to London. Training took place a few months prior to the start of the study and consisted of two residential courses for the Manchester lay trainers and London lay trainers separately. Post training, 60% of the lay trainers did not actively participate in the study. Training costs for practice nurses were £5600 (£2800 for each site), with a mean training cost of £18 per patient (Manchester £34, London £14). There was no need for re-training within the trial period, re-training would be outwith the time horizon of this study.

### Delivery

The lay educators received an hourly rate for consultations of £8. In contrast, practice nurse hourly rates were £32 (London) and £28 (Manchester). Table 2 shows that lay educators completed more consultations than the nurses (461 versus 325) and had lower costs for consultations per patient when compared with practice nurses.

### Unscheduled healthcare

The outcome unscheduled healthcare and the associated types of healthcare utilisation were also costed . Table 3 shows that the unscheduled health care usages in both arms are very similar with a total number of 178 events in the lay educator arm and 177 events in the practice nurse arm. There were more hospital admissions in the lay educator arm (109 versus 88, p = NS), and lower levels of GP consultations in the lay educator arm compared to the practice nurses (48 versus 62, p = NS). Costs were lower for lay educators for hospital admissions and A&E attendances. Table 3 shows the mean unscheduled healthcare costs were £161 for practice nurses compared to £135 for lay educators.

**Table 3:** Unscheduled healthcare usage and costs

		**Practice nurse n = 205**			**Lay educator n = 213**		
	**Unit Cost**	**Total number of events**	**Median, range per patient**	Mean cost per patient, (SD)*	**Total number of events**	**Median, range per patient**	Mean cost per patient, (SD)*
Hospital admission	£932 ^1^	88	0, 0-6	£123 (£596)	109	0, 0-3	£92 (£448)
GP Attendance (per surgery consultation lasting 12.6 minutes)	£30 ^2^	62	0, 0-1	£12 (£32)	48	0, 0-6	£15 (£34)
A&E attendance (standard attendance)	£61 ^3^	27	0, 0-10	£18 (£64)	21	0, 0-6	£13 (£46)
ICU hospital stay^¥^	£1470 ^4^	1	0, 0-1	£7 (£105)	2	0, 0-1	£14 (£145)
Total number of unscheduled healthcare events				**£161 (£718)**			**£135 (£553)**

^*^The mean costs per patient were calculated at an individual patient level, e.g. patients may have had more than one hospital admission or GP attendance during the study as shown by the median and range.

^**¥**^**ICU hospital stay has not been included in the unscheduled healthcare event as it has already been included in the hospital admission count.**

^**1**^**(Mean 4 days length of stay) National schedule of reference costs 2004–2005.**

^**2**^**PSSRU 2004–2005.**

^**3**^**National tariff 2005/2006.**

^**4**^**National schedule of reference costs 2004/2005.**

Table 4 shows the costing analysis outcome where there were significant differences (p < 0.001) in staff training costs between the two groups, with lay educators (£36 per patient) costing significantly more to train than the nurses (£18 per patient). The costs of delivery of the intervention consultations were significantly higher for nurses (£23 per patient) when compared to lay educators (£8 per patient). Unscheduled healthcare costs were slightly lower (not significant p = 0.681) for the lay educator arm (per patient) at £135 compared to the nurses at £161. Overall costs for all consultations, training and unscheduled healthcare showed no significant differences between the groups (nurse = £202 versus lay educator = £178, p = 0.707).

**Table 4:** Cost balance sheet

**Mean cost in £ sterling per patient (SD)**	**Practice nurse (n = 205) Mean per patient (SD)**	**Lay educator (n = 213) Mean per patient (SD)**	**Difference, [95 % CI]**	P-value*
Staff training costs	£18 (£9)	£36 (£20)	-£17 [−£20, -£14]	p < 0.001
Healthcare delivery Consultation costs	£23 (£23)	£8 (£6)	£15 [£12, £18]	p < 0.001
***Total cost of training and healthcare delivery costs***	**£41 (£24)**	**£44 (£21)**	**-£2 [−£7,£2]**	**p = 0.287**
Unscheduled healthcare costs	£161 (£718)	£135 (£553)	£26 [−£97, £149]	p = 0.681
***TOTAL COSTS***	**£202 (£717)**	**£178 (£554)**	**£24 [−£100, £147]**	**p = 0.707**

* Independent student *t test.*

### Scenario analysis

Scenario analyses were undertaken to investigate the uncertainty in the study data and to relax some of the baseline models stringent criteria (Table 5). The total cost of the intervention if the practice nurses and lay educators had fulfilled the protocol and given all patients two consultations and 4 telephone consultations (Scenario 1) would have increased the costs to £241 for practice nurses and £186 for lay educators but still with no significant differences between the study arms (£54). There were some difficulties adhering to the protocol within the trial, where both nurses and lay-trainers did not always manage to give each patient two face to face consultations and four telephone consultations. Lay trainers were part-time and nurses still had a full clinical load, which impacted on time available to see the study patients. Scenario 2 builds on the first scenario but only 50% of the consultation costs for lay educators are used to examine the impact of the large majority of lay advisers who did not claim back for their time. When the cost of building in extra time for the telephone consultations was added the cost rose to £260 for practice nurses and £191 for lay educators (Scenario 3).

**Table 5:** Scenario analysis - Total cost (training, intervention and unscheduled healthcare usage)

	**Practice nurse (n = 205)**	**Lay educator (n = 213)**	**P-value**
	**Mean (SD)**	**Mean (SD)**	
Baseline analysis	£202 (£717)	£178 (£554)	p = 0.707
***Scenario 1****:* Protocol adherence	£241 (£718)	£186 (£554)	p = 0.385
***Scenario 2:*** Protocol adherence and 50% of consultations costs for lay educators	£241 (£718)	£179 (£554)	p < 0.001
***Scenario 3****:* Protocol adherence, and extra preparation time for telephone consultations (20 minutes for each telephone consultation)	£260 (£718)	£191 (£554)	p = 0.279
***Scenario 4****:* Protocol adherence, 50% shorter consultations for nurses for first and second consultations, and extra preparation time for telephone consultations (20 minutes for each telephone consultation)	£240 (£719)	£191 (£554)	p = 0.437

Scenario 4 examined the effect of adhering to the study protocol, reducing the length of nurse face to face consultations and adding in extra time for the telephone consultations, costs were £240 for the practice nurses and £191 for lay educators. There were no significant differences between the intervention and control groups for any of the scenario analyses except Scenario 2 (p < 0.001).

## Discussion

Within trial analyses have shown that there were no significant overall cost differences between nurses and lay-trainers. However there were significant differences between the two groups with regards to the cost of training (significantly higher for lay educators) and the cost of delivering the trial consultations (significantly lower for lay educators). There was a cancelling out effect of the two components of the intervention with lower costs for nurses in the training part and lower costs for lay educators in delivering the consultations, however there was no overall difference in effect (frequency of healthcare utilisation events). Although the asthma trainers were more expensive to train per patient the trial results show that they can deliver asthma consultations at a much lower cost. The provision of robust training is essential to the safety, acceptability to practices and confidence of the lay educators. The lay educators were providing direct care and substituting for a practice nurse.

In this analysis we have not included the cost of basic nurse training. We set out to assess the additional extra cost of skilling either a lay person or a nurse to competently undertake an asthma review. Hidden costs such as the cost of mentoring and supporting the practice nurses and lay educators in practices were not explored, although it was noted that extensive support was given at the beginning of the trial. To extrapolate this study to real life the lay educators would need some form of mentoring within their practices. Due to the under-recruitment in the Manchester arm of the study the results, whilst showing no differences in outcomes between the two arms, did not actually have sufficient power to demonstrate equivalence.

The cost of follow-up telephone consultations incorporated staff time, but did not include the administrative cost of calls. These costs would have been similar in both nurses and lay educators. Since this study has been carried out there has been an increase in tele-health and perhaps using texts or reminders may have increased the numbers of telephone consultations carried out. The increase in mobile phones usage in comparison to landlines would also be interesting to investigate further.

As discussed in the original trial using lay educators to deliver health care, the study had a number of logistical problems. Several of the trainers dropped out of the study shortly after training, significantly contributing to the training costs. This was in part due to a delay in the trial start-up. The asthma trainers were not contractually employed and the training course was a “one-off” making it difficult to re-recruit new trainers. Updating of training and training of new staff should be considered if lay educators are rolled out into clinical practice. Prior to the trial, and contrary to expectations, very few of the practice nurses had had any additional asthma training (only one of the forty-six nurses had an Asthma diploma). This low rate of acquisition of training to permit nurses to competently undertake additional tasks such as asthma reviews may or may not be representative of other parts of the UK [28]. Due to competing pressures for time many practice nurses found it difficult to conform to the trial protocol, as is seen with the lower completed consultation rates compared with the lay educators. The lay educators for example, were more successful than nurses in contacting patients and persuading them to attend for review and undertook more telephone follow ups. In reality it is likely that the nurses did not adhere to the recommended time durations for consultations which would have reduced their costs as we have considered in our scenario analysis, although it is likely that lay educators might also “speed up” with time. Certainly unit costs for the lay educators could be expected to be reduced in real life, since within the trial, the lay educators often travelled to a practice to see only one or two patients randomised to them, whereas a lay educator working full time and in one location and preferably across practices could be more effective.

These scenarios were devised assuming that changes in intervention delivery would not impact on unscheduled health care utilisation. Scenario 1 is the most likely scenario to have an impact of the unscheduled healthcare utilisation. However full protocol adherence was in both arms of the study and improvement in outcomes was similar and therefore no significant difference is seen.

 This trial, if completed according to protocol would have represented one of the largest trials of review and self management support ever undertaken. The difficulties with recruitment in Manchester, accompanied by problems with Ethics Committee approvals and delays in utilising lay educators newly acquired skills, led to a situation where there was drop out of educators, practices and nurses and full equivalence of intervention was intimated if not quite proven. However few seeing these results can doubt that in one prescribed area of competency, well trained lay educators have been shown to be capable of delivering healthcare in a manner comparable to traditional care, which in the UK is by primary care based Practice Nurses. A lay-led asthma self management education package may not be possible in all healthcare systems, particularly where patients expect to receive care from a medically qualified individual or where there is a high level of medical dominance.

### Practicalities of implementing lay educators into clinical practice

This study has shown that there was no difference in the cost of unscheduled healthcare between the nurses and the lay educators. It would not be possible to replace nurses completely within general practices as only a small proportion of their time is spent on patients with asthma, one study has shown that this is as little as 7% of their time [29]. Potentially we could free up clinical time to see other patients and perform other duties but it would not be in effect a cost saving because of additional spending on lay services while nurses are still employed.

It should be considered that using lay educators would incur extra costs providing an additional service albeit at a significantly lower cost than nurses. Other factors should also be considered, we found in the original study that there was a significant number of lay educators who dropped out before and after training and high levels of practice nurse turnover. Lay educators would have to appropriately recruited and trained to ensure training costs are not wasted. Training would need to be reinforced and perhaps repeated annually to ensure adequate skill levels which would add to the running costs. Ultimately lay educators would need to be reliable and as members of the public may be more of an unknown quantity than already employed nurses.

Lay educators will also need to be employed on a formal contract and would need continued mentoring and guided support to avoid isolation, both of these issues were raised during the trial. It is crucial that the role of the lay educator is well defined and the employment contract is well defined as shown by Brown *et al.*[21]. In this study lay educators were responsible for claiming back the costs for their time at £8 per hour. The employment contract was not well defined and it is difficult to perceive whether individuals were acting as an expert member of staff or volunteer. This has implications on the success of the individual at delivering the outcomes and more research needs to be carried out on the role, expectations and effectiveness of expert patients in trials and clinical practice as well as the roll-out cost implications. It would be considerably cheaper to use lay educators as a volunteers but further work on the implementation of lay educators into “real-world” clinical practice needs to be carried out to ensure this is the best option.

## Conclusions

There were no significant differences in costs of training or delivery, no significant differences in costs of unscheduled healthcare and no overall significant differences in costs. However there are wide confidence intervals with both positive and negative values which suggest uncertainty. Results from this study show that lay trainers cost more to train up to deliver care compared to nurses but their employment costs were less. As the outcomes appeared to be similar to those of nurses further work is needed to see how we can use them more effectively to ensure their higher training costs are used to maximise delivery of lower cost care. Further consideration and exploration of the potential role of lay educators within the NHS is needed.

## Competing interests

The authors declare that they have no competing interests.

## Authors' contributions

All authors contributed to the design and development of the costing study. KB and NJR carried out the data analysis. All authors were involved in the drafting of the manuscript.

## Pre-publication history

The pre-publication history for this paper can be accessed here:

http://www.biomedcentral.com/1471-2466/12/52/prepub
